# Duration of In-hospital Stay for Elective Neurosurgical Procedures in a Tertiary Care Hospital

**DOI:** 10.7759/cureus.15745

**Published:** 2021-06-18

**Authors:** Bilal Khan, Usman Haqqani, Sajjad Ullah, Saima Hamayun, Zohra Bibi, Khalid Khanzada

**Affiliations:** 1 Neurosurgery, Medical Teaching Institution/Lady Reading Hospital, Peshawar, PAK; 2 Neurosurgery, Qazi Hussain Ahmed Medical Complex, Nowshehra, PAK; 3 Neurosurgery, Medical Teaching Institution/Khyber Teaching Hospital, Peshawar, PAK; 4 Medicine, Khyber Medical College, Peshawar, PAK; 5 Psychiatry, Medical Teaching Institution/Lady Reading Hospital, Peshawar, PAK; 6 Neurosurgery, Ibrahimi Medical Center, Peshawar, PAK

**Keywords:** in-hospital stay, duration, elective, neurosurgical procedures, tertiary

## Abstract

Objective: Public hospitals have fixed days with allotted time slots during which to perform neurosurgical elective cases. If emergency operations or other events preempt these scheduled time slots, the patient remains hospitalized, waiting in queue for a new time slot. We conducted this study to determine the number of days patients remained admitted waiting for elective cases in a tertiary care public hospital, which operates on fixed days.

Materials and methods: This cross-sectional study was conducted in the Department of Neurosurgery Unit B, Medical Teaching Institution (MTI) - Lady Reading Hospital (LRH), Peshawar. We reviewed the admission charts and discharge slips of all patients who were admitted and underwent operations between September 2018 and August 2019. A form was made and was completed with each patients' records like age, gender, number of days spent preoperatively and postoperatively and the total duration of stay, indication for surgery (spinal, cranial, peripheral nerve), etc. Patients who had undergone elective neurosurgical procedures were included while those who had undergone emergency surgeries or had expired during the hospital stay, had been discharged or referred to other centers were excluded from the study. All the data were entered into the statistical software SPSS version 22 (IBM Corp., Armonk, NY) and were converted into tables and charts.

Results: A total of 1818 patients were admitted/discharged during the study period, and of them, 823 patients were admitted for elective neurosurgical procedures. There were 601 (73.7%) males and 222 (26.3%) females with a male to female ratio of approximately 3:1. The age range was from 09 days to 72 years and was further subdivided into six groups. The procedures were broadly divided into cranial, spinal, related to hydrocephalus (HCP)-related, and miscellaneous. Cranial procedures comprised of surgeries for brain tumors, transsphenoidal operations, vascular procedures for aneurysms, and nerve decompressions, and they comprised about 29.43% (n=244) while spinal procedures accounted for 317 (36.63%) procedures, the rest were related to HCP and miscellaneous. Preoperative and postoperative stay durations were calculated and then added to determine the total stay durations and were further stratified for the specific procedures and categorized into days and weeks. About 58.26% (n=143) of cranial cases, and 156 (49.36%) of spinal cases, 37.57% (n=65) of HCP-related cases, and 36.66% (n=41) of cases in the miscellaneous group had a duration of stay between eight days to more than three weeks.

## Introduction

Healthcare is a major problem worldwide, though there is a lot of difference between that of a developed and developing nations, because of the latter poverty and low income of people [1]. The preferred mode of treatment of patients in the former are the public institutions because of the lack of insurance and low socioeconomic status of the population, and free of charge access of public hospitals, yet many other factors that impede timely access to healthcare institutions [1]. Apart from the prolonged waiting period for elective surgical procedures, prolonged length of stay (LOS) is defined as an inpatient stay that exceeds the expected LOS for a specific procedure. Thus, aside from prolonging the wait for elective surgeries, prolonged LOS results in patients occupying hospital beds and prolonging the wait times for elective admissions, the latter also leads to under-utilization of hospital resources [2,3]. Since elective surgeries are performed more frequently than emergency surgeries, in most of the institutions the operating rooms are on fixed days and the patient has to wait their turn for the procedures, while they remain admitted in the hospital [4,5]. The American College of Surgeons' (ACS'), National Surgical Quality Improvement Program (NSQIP) is a uniform database accessible to reviewers for assessment of LOS for the above elective procedures [6].

Neurosurgical surgeries are prolonged and require a proper assessment prior to surgeries and require further postoperative support in the intensive care unit [3,6,7]. These issues, along with the fact that in most public sector hospitals, the elective cases are performed on specific days, further add to LOS prolongation for the patients, who remain admitted in the ward awaiting surgeries. We conducted this study to determine the average LOS for patients who were admitted to the neurosurgical unit for various elective procedures, and to compare it with the standards being observed worldwide.

## Materials and methods

The cross-sectional study was conducted in the Department of Neurosurgery Unit B, MTI-Lady Reading Hospital (LRH), Peshawar, Pakistan. We reviewed the admission charts and discharge slips of all patients who were admitted and underwent elective neurosurgical procedures between September 2018 and August 2019. A form was designed and was filled out for each patient with their age, gender, primary indication for surgery, duration of stay (preoperative, postoperative, and total), and indication for surgery (spinal, cranial, peripheral nerve), etc. Data were further stratified by forming subgroups for age, and the duration of stay. Patients undergoing elective neurosurgical procedures were included while those who had undergone emergency surgery or had expired during the hospital stay, and those discharged and referred to other centers were excluded from the study. All the data were entered in the statistical software SPSS version 22 (IBM Corp., Armonk, NY) and were converted to tables and charts. Means were calculated for numerical variables like age, pre and postoperative durations, while frequency and percentages were determined for categorical variables like gender and type of surgery.

## Results

**Figure 1:**
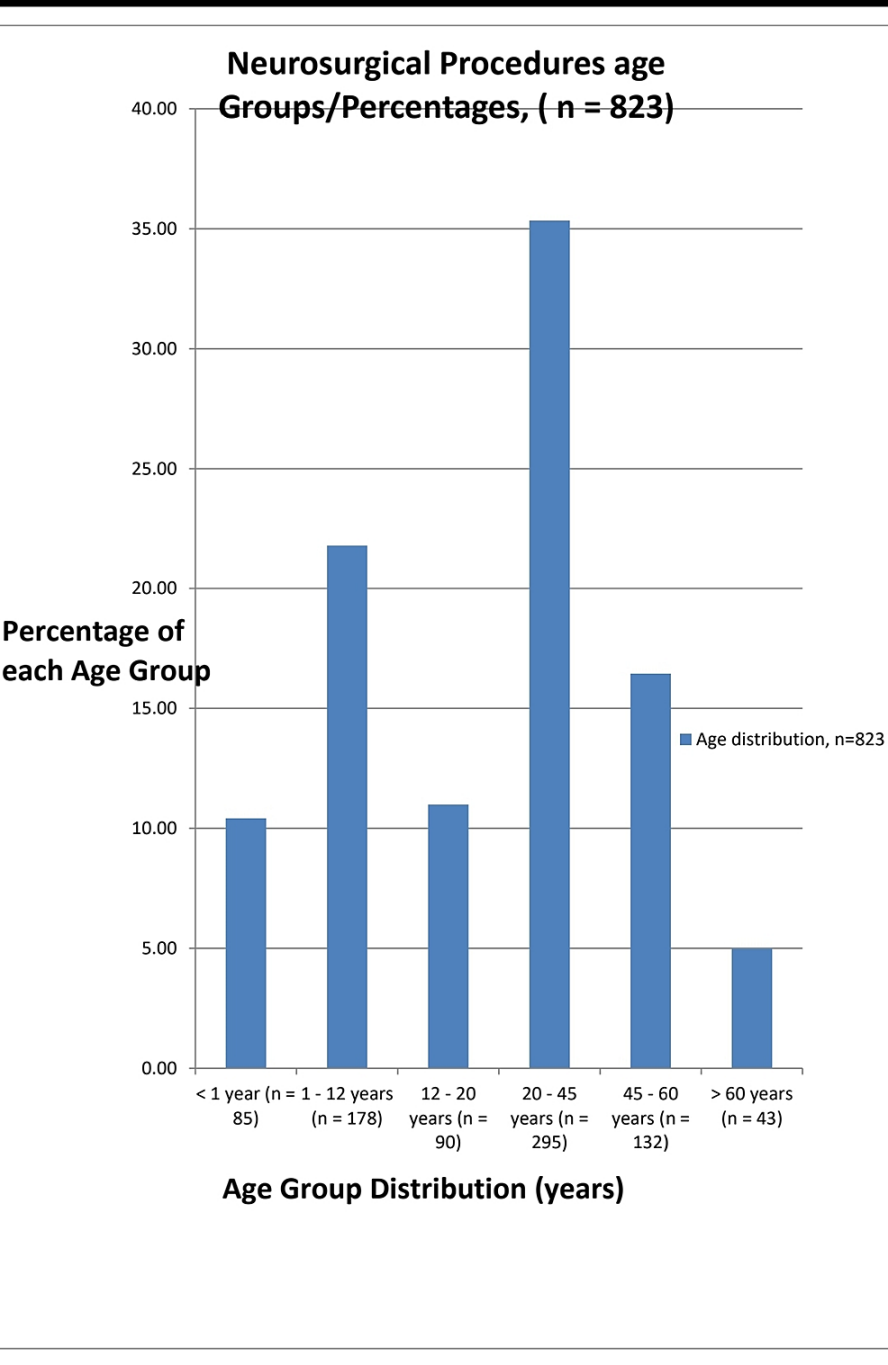
The distribution and relative percentages of each age group admitted for elective neurosurgical procedures.

A total of 1818 patients were admitted/discharged during the study period and of these 823 patients had undergone elective neurosurgical procedures. There were 603 (73.7%) males and 220 (26.3%) females with a male to female ratio of approximately 3:1. The age range was from 9 days to 72 years and was further subdivided into six groups (Figure 1).

**Figure 2:**
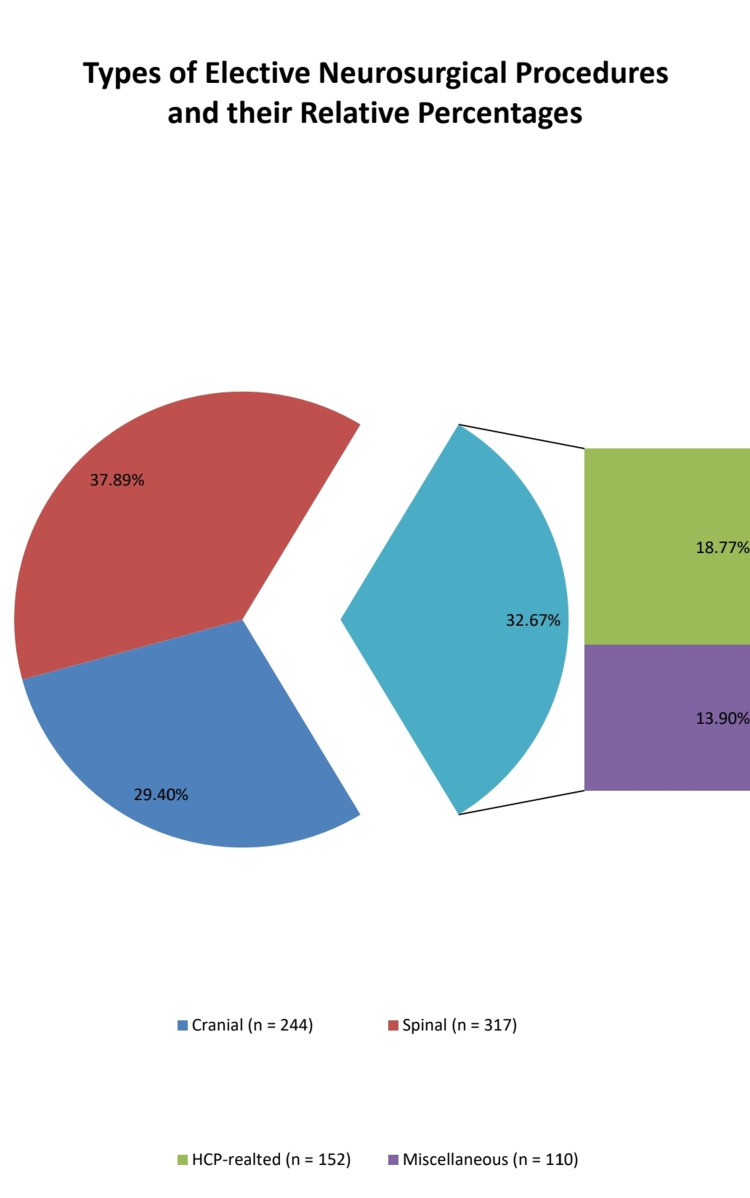
The pie chart depictions of the categories of elective neurosurgical procedures and their relative percentages. HCP: hydrocephalus.

The elective neurosurgical procedures were divided into cranial, spinal, hydrocephalus (HCP)-related; as HCP represented a substantial number of patients and the procedure related to it was mostly ventriculoperitoneal shunt which also has an abdominal component and does not require formal craniotomy either, and miscellaneous (dressing and debridement, myelomeningocele repair, and cerebrospinal fluid (CSF) leak repairs). Cranial procedures consisted of surgeries for brain tumors, transsphenoidal operations, vascular procedures for aneurysms, and cranial nerve decompressions, which together made about 29.43 %(n=244), while spinal procedures accounted for 317 (36.63%) cases and were also subdivided into simple decompressions and those requiring instrumentation, the rest were related to HCP and miscellaneous (Figure 2).

**Figure 3:**
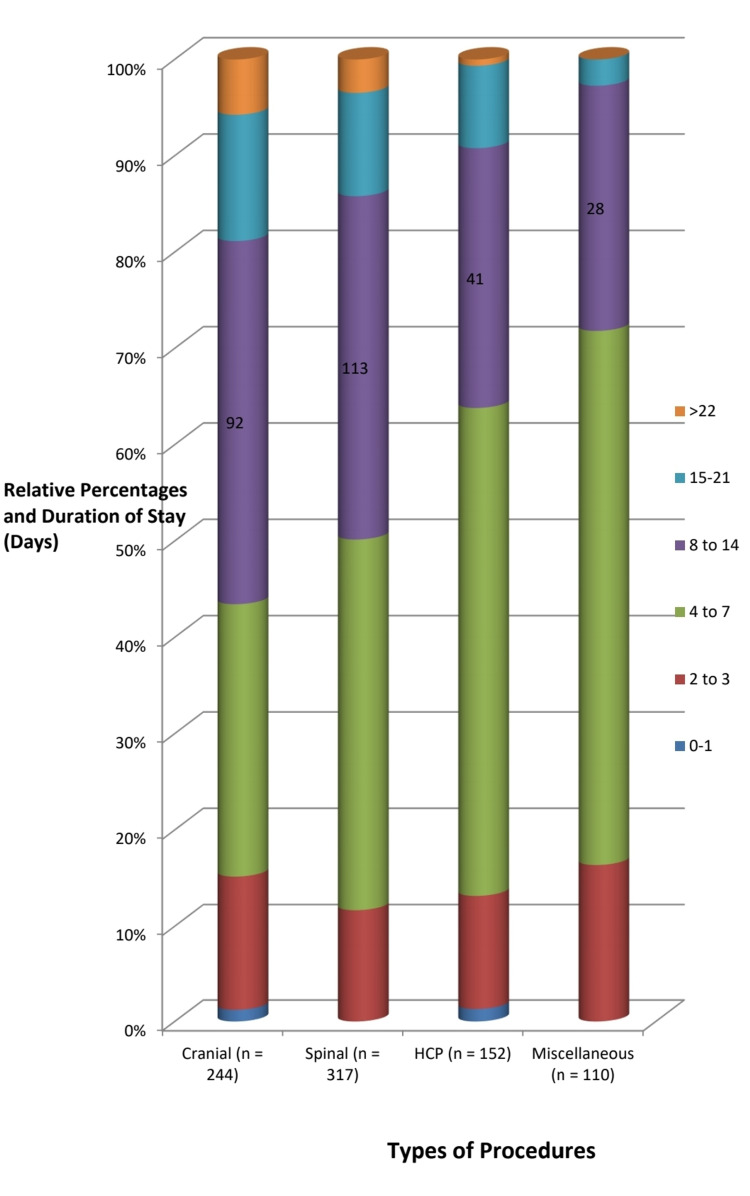
The breakdown of the length of stay of patients within each category of elective neurosurgical procedures.

The in-hospital stay was calculated as preoperative, postoperative, and total duration, and was further stratified for the specific procedures and categorized into days and weeks. The mean duration of hospital stay was 6.5 days with a standard deviation (SD) of ±5 days, the mean preoperative hospital stay was 4.5 days (SD of ±4), and the mean postoperative hospital stay was 3.4 days (SD of ±2.9). The duration of stay was between 8 days and >3 weeks for 58.26% (n=143) of cranial, 156 (49.36%) of spinal, 37.57% (n=65) of HCP-related, and 36.66% (n=41) of miscellaneous cases. A further delineation of the duration of stay related to the procedures is given in Figure 3.

**Figure 4:**
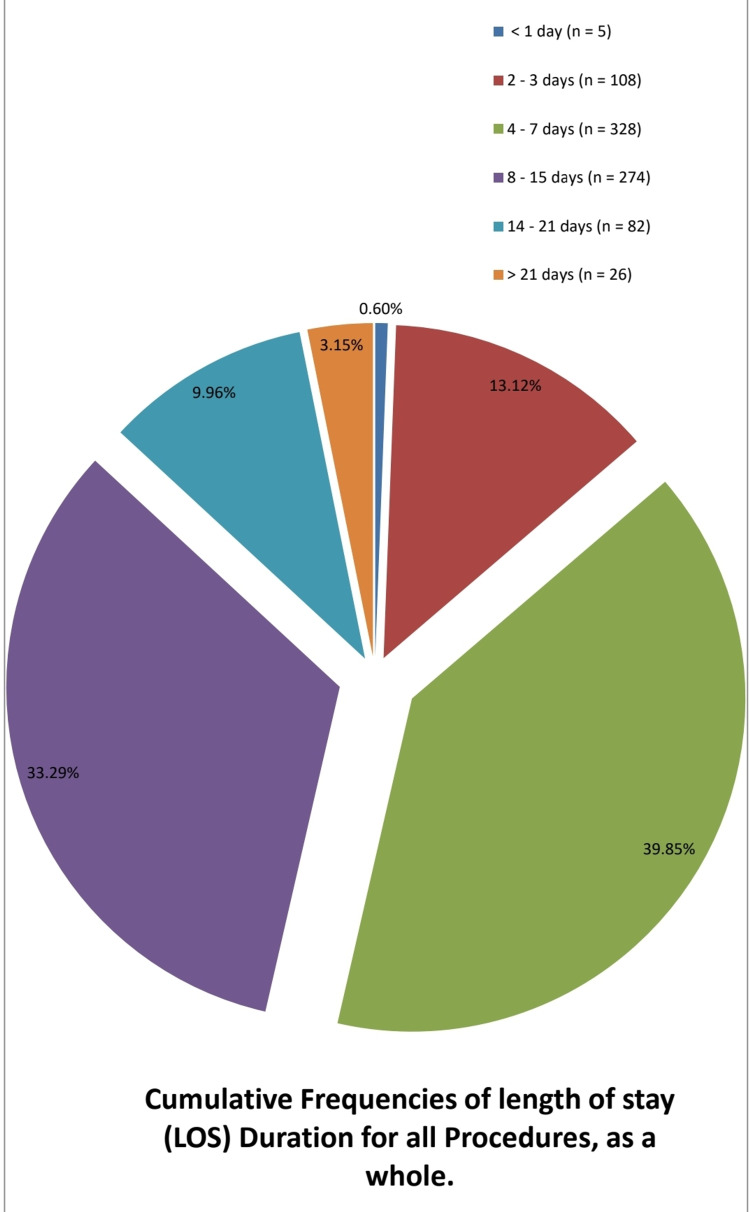
Image depicts the cumulative length of stay for all patients irrespective of the types of elective neurosurgical procedures.

Almost half of all patients admitted for any procedures had a LOS of more than one week regardless of the type of surgery (Figure 4).

The mean duration of stay for the preoperative and postoperative periods and also the total duration of hospitalization are presented in Table 1.

**Table 1:** The duration of stay for all neurosurgical procedures, as pre-op, post-op, and total duration of the length of stay.

Procedures	Pre-op (mean days)	Post-op (mean days)	Total (mean days)
Craniotomy	6.86	4.84	11.73
Spinal procedures	6.10	1.84	8.97
Hydrocephalus related	4.20	3.46	7.72
Miscellaneous	4.5	3.8	7.9

We had four operating tables available for two days a week and surgical time allocated from 8:00 am to 4:00 pm, which starts at about 8:40 am, and the cut-off time for anesthesia is 1:30 pm. Thus, we can calculate the total number of cases done per day, per week, and per operating table per day on the list as shown in Table 2. Sometimes major cases like craniotomies for brain tumors are done once per table per day, but minor cases are done three to four per table per day and a major case can be accompanied by a minor case, sometimes the OT days occur on public holidays, two cases are performed however on each operating table. Furthermore, our admission days were on Tuesday and Thursday, and very seldom is a patient admitted on these days had their surgery the next day, i.e., Wednesday and Friday, is also a prayer day and no surgeries are performed after 1:00 pm. This reflects that there were factors other than being postponed due to an unprepared case, such as the lack of the allocated operating time available which results in the prolonged stay in hospital. Our study lacked the comprehensive evaluation of reasons for being postponed resulting in prolonged LOS because of its retrospective nature and the inherent difficulties of determining reasons for delay after the fact.

**Table 2:** The number of cases operated and workdays, and also the number of cases operated per day and per week.

No. of cases	No. of tables	No. of OT days per week	No. of cases per week	No. of cases per day	No. of cases per table per day
823	04	02	15.82	7.91	1.97

## Discussion

Hospital LOS for elective procedures is an important factor, which has wide implications on the hospital resources to be utilized properly. Outcomes and patient satisfaction are to a great extent dependent on timely access to and availability of these elective services. Since surgical procedures represent approximately 40% of the hospital costs, and elective surgeries represent more of the burden than the emergency services, the duration of stay has a profound impact on the total costs incurred upon the patient [2,5-7].

This study was conducted to determine the duration of stay for various elective neurosurgical cases in our department and to compare each subgroup's LOS with the standard LOS. After reviewing the charts over a year and stratifying the data, we found a significant difference in the male to female ratio, i.e., 3:1. Our general ward had 14 female beds and 18 male beds, but no proper explanation could be justified, except that small children and babies of either sex were kept in the female wards with their mothers. No such significant difference in gender was found in the paper by Asenjo et al. [5]. In a series by Collins et al., however, the number of males were higher than females, undergoing various surgical procedures including spinal procedures like laminectomy, in which the percentage of male patients was more than 97.9% [8].

The procedures were categorized into cranial, spinal, hydrocephalus-related, and miscellaneous. The cranial procedures had a LOS of 11.73 days. There has been no standard LOS available for cranial procedures, and only 40% of these cranial procedures had LOSs <7 days. In a review of 11,500 patients undergoing operations for brain tumors, Dasenbrock et al. showed that the LOS for craniotomy was <8 days in 72.72% of patients [6]. The average LOS was 4.7 to 6.0 days in a series by Prentice and Pizer [7] for various cranial procedures, while in our case it was 11.73 days, which is about five to seven days longer. However, the reason for our delay can not be elucidated, but nonetheless, the difference is high. It has been shown in our series that the preoperative duration of stay was significantly higher than the postoperative duration, i.e., seven days versus five days, as shown in the Results section, and it shows that the fixed operative days somehow has an impact on the patients LOS, though we did not determine any other significant reason for our delay.

While the standard LOS for most spinal procedures is about five days [8], only about 40% of patients in our study had a LOS of equal to or less than five days, and patients even with simple lumbar discectomies had a prolonged duration of stay, i.e., >5 days. In a study, Collins et al. have shown a duration of stay >8 days in about 30% of cases patients undergoing lumbar procedures, and the median LOS was five days, but that was also because of the associated comorbidities like diabetes, myocardial infarction, renal failure, and congestive heart failure [8-10]. Though we did not study all these factors, they cannot account for more than 60% of patients having a prolonged hospital stay, as most of these patients undergoing spinal surgeries were in a good state of health. As a general observation from the neurosurgical private practice at the same institution, where approximately 700 lumbar surgeries are performed annually, the standard LOS for lumbar surgeries is one day and it very rarely exceeds more than three days for a healthy patient. Even in a wide range of surgeries, the complication rate is reported to be between 2.4% and 3.6%, as shown in the study by Smith et al., in his series where a total of 9692 lumbar decompressions (LDs), 6735 ACDFs, and 10,329 lumbar spinal discectomies (LSDs) were identified, with overall complication rates of 3.6%, 2.4%, and 7.0%, respectively, i.e., 2.4% was the complication rate for ACDFs and 3.6% was for lumbar discectomies [11,12], which cannot account for more than 60% of the patients having a prolonged LOS. Even in centers, ACDFs and lumbar laminectomies are performed as outpatient surgery, and there is no LOSs available for comparison [10], and as stated, compared to the standard private practice's LOS at the same institution even five days are considered a prolonged LOS.

The LOS for HCP was 7.73 days. Since HCP is a result of many brain disorders and it is difficult to measure LOS for it alone, and study by Nigim et al. [13] showed that mean LOS was five days for primary HCP, while Khan et al. [14] showed that the mean and median duration of hospital stay for patients was 11.7 days and five days, respectively, however, most of the cases were adults and were having many comorbidities, and also HCP was secondary to other brain problems like tumors. Our study was limited as we do not know how many of the shunt cases were complicated. However, the LOS for various disorders like myelomeningocele, duroplasty, and debridement procedures had been quite variable, as these were mostly complicated procedures and the hospitalized patients were retained for antibiotic therapy and wound care. A study by Onjel et al. showed that the LOS ranged from 13 to 42 days depending upon whether the patients had any complications or not [15].

As noted above and from the results, it was obvious that the preoperative duration was quite prolonged as compared to the postoperative duration, the latter of which was significantly shorter, especially for spinal disorders. Although this study has limitations as we did not mention the causes for the delay and the reasons for patients not being on the list or lack of operative time, this usually represents a small proportion. We can deduce that the fixed operative days had an impact on the prolonged LOS in the majority of patients. This needs to be further validated and verified by prospective studies.

## Conclusions

It has been observed that elective neurosurgical procedures with fixed operative days have a prolonged LOS, and it was evident by the prolonged preoperative duration, which was significantly higher than that of the postoperative duration. Even, the total duration was significantly higher as compared to the that listed and reported in the literature, for many procedures like simple lumbar discectomies, and as stated, the waiting period in the form of preoperative duration could be the reason for this prolonged LOS. Since it was a retrospective study and many factors cannot be studied, which makes it a limited study, the large differences in the LOSs compared to the standard LOSs is quite significant, with a major contribution from the prolonged preoperative stay, and as such the policy of fixed days need to be reconsidered.
